# Editorial: Rising stars in neurorobotics 2021

**DOI:** 10.3389/fnbot.2022.1109498

**Published:** 2022-12-16

**Authors:** Giacomo Valle

**Affiliations:** ^1^Department of Organismal Biology and Anatomy, University of Chicago, Chicago, IL, United States; ^2^Department of Health Sciences and Technology (D-HEST), IRIS, ETH Zurich, Zurich, Switzerland

**Keywords:** neurorobotics, neuroprosthetics, neuromodulation, embodiment, neurorehabilitation, prosthetics

Recently, technological breakthroughs in neuro-robotics were translated into a more complete interfacing of humans and machines, targeting the residual nerves or central nervous system of neurologically disabled individuals. In current neuroprosthetic devices, the functionality and the embodiment of the technology are crucial topics for their wider adoption (Raspopovic et al., 2021). Studying the functional, health and cognitive implications of using neurorobotic devices and brain-computer interfaces in different clinical populations become especially relevant in this moment of human history, when many growing companies such as Neuralink, Inbran Neuroelectronics, Synchron and others, are going into human certified applications with the need of a better understanding on the effects of interfacing human nervous systems with robots (Valle, 2019). In this Research Topic, rising stars scientists in the field of neurorobotics tackled important challenges for the development of the next generation of neurotechnologies.

Indeed, the field of prosthetics has been revolutionizing by the adoption of implantable neural interfaces connecting the human nervous system of patients with the robotic limbs (Raspopovic et al., 2021). In the last decades, unprecedented development of neurotechnology for human neurorehabilitation, personalized use, and cognitive enhancement has been observed. Novel brain-computer interfaces (BCIs) have been proposed to address a variety of therapeutic improvements and challenges, such as sensory-motor restoration, improving decision-making and modulating mood disorders. In this direction, Handelman et al. implemented a collaborative shared control strategy to manipulate and coordinate two Modular Prosthetic Limbs (MPL) for performing a bimanual self-feeding task. In particular, a human participant with microelectrode arrays in sensorimotor brain regions provided commands to both MPLs to perform the self-feeding task, which included bimanual cutting. This demonstration of bimanual robotic system control *via* a BCI in collaboration with intelligent robot behavior has major implications for restoring complex movement behaviors for those living with sensorimotor deficits. Since this new generation of implantable BCI will be able to intimately interact with the human brain, a proactive ethical approach is needed to ensure that these new technological developments go hand in hand with the development of a sound ethical framework (Valeriani et al.). To this aim, Valeriani et al., propose a foresight map that classifies ethical and societal risks based on the time frame in which they are expected to emerge and generate societal concern.

Interestingly, the restoration of sensory-motor functions, exploiting neurorobotic device directly interfacing the human nervous system, have relevant effects on also cognitive aspects related to the user. For this reason, scientists in the field of neuroprosthetics require dependable measures of ownership, body representation, and agency to quantify the sense of embodiment felt by patients for their neuroprosthetic limbs. Segil and Graczyk reviewed the outcome measures used in the literature to identify the senses of ownership, embodiment, and agency. Advances in the ability to quantify the embodiment of prosthetic devices have far-reaching implications in the development of sensory-enabled prosthetic limbs as well as promoting a broader understanding of ourselves as embodied agents. Furthermore, Risso and Bassolino suggested that neurorobotics can make an important contribution to the study of mental body representations. This study illustrates an emergent multidisciplinary perspective combining the neuroscience of body representations and neurorobotics to understand and modulate the perception and experience of one's own body. In particular, the authors suggest that neurorobotics can improve experimental rigor introducing new experimental conditions and support the rehabilitation of the distorted body perceptions.

With the development of novel neurorobotic devices with the aim of improve patients assistance and rehabilitation, there is also an increasing need for a fast testing and validation. For example, the validation of myoelectric prosthetic control strategies for individuals experiencing upper-limb loss is hindered by the time and cost affiliated with traditional custom-fabricated sockets. Hansen et al. present a multi-user, low-cost, trans-radial, functional-test socket for short-term research use that can be custom-fit and donned rapidly, used in conjunction with various electromyography configurations, and adapted for use with various residual limbs and terminal devices. The development of this multi-user, trans-radial, functional-test socket constitutes an important step toward increased end-user participation in advanced myoelectric prosthetic research. Importantly, the socket design has been open-sourced and is available for other researchers.

In addition, the use of neurorobotic devices could help in assessing and treating also degenerating neurological disease as multiple sclerosis (MS). Indeed, Pierella et al. show how to quantify upper limb motor deficits in asymptomatic MS subjects with a robot-based assessment including performance and muscle synergies analysis. Asymptomatic MS subjects generated less smooth and less accurate cursor trajectories than control subjects in controlling a force profile, while the end-point error was significantly different also in the other environments. Moreover, the EMG analysis revealed different muscle activation patterns in MS subjects when exerting isometric forces or when moving in presence of external forces generated by a robot. These results suggested that a task requiring to control forces against a rigid environment allows better than movement tasks to detect early sensory-motor signs related to the onset of symptoms of multiple sclerosis and to differentiate between stages of the disease.

In addition to neurotechnologies for improving sensory-motor recovery, the neurorobotic field is proposing innovative approaches, based on neuromodulation, to target the autonomic nervous system generating a novel branch of medicine called bioelectronics (or electroceuticals). Although pharmacological interventions are currently the mainstay of treatment, neurostimulation offers a potentially effective and safe alternative, capable of providing rapid adjustment to short-term variation and long-term decline of physiological functions. On this topic, Rowald and Amft believe that the future of neurostimulation strongly depends on personalizable computational tools, i.e., Digital Neuro Twins (DNTs) to efficiently identify effective and safe stimulation parameters. The authors outline how DNTs will pave the way toward effective, cost-, time-, and risk-limited electronic drugs with a broad application bandwidth.

All in all, these works constitute an important landmark for the fields of neurorobotics, human-machine interfacing, and neurorehabilitation opening exciting and realistic perspectives for further developments of effective neuro-robotic devices that will drastically change life of people with disabilities.

## Author contributions

The author confirms being the sole contributor of this work and has approved it for publication.
